# Long*-*term analysis of multimodality treatment outcomes and prognosis of esthesioneuroblastomas: a single center results of 138 patients

**DOI:** 10.1186/s13014-020-01667-4

**Published:** 2020-09-18

**Authors:** Meng Sun, Kai Wang, Yuan Qu, Jianghu Zhang, Shiping Zhang, Xuesong Chen, Jingbo Wang, Runye Wu, Ye Zhang, Junlin Yi, Jianping Xiao, Guozhen Xu, Xiaodong Huang, Jingwei Luo

**Affiliations:** Department of Radiation Oncology, National Cancer Center/ National Clinical Research Center for Cancer/Cancer Hospital, Chinese Academy of Medical Sciences and Peking Union Medical College, Beijing, 100021 China

**Keywords:** Esthesioneuroblastoma, Treatment strategy, Survival outcomes, Prognostic factors

## Abstract

**Background:**

The aim of this study is to evaluate the efficacy of different treatment strategies and the potential prognostic factors of esthesioneuroblastoma (ENB).

**Materials and methods:**

Between April 1984 and December 2018, 138 patients with non-metastatic ENB were retrospectively analyzed. The treatment modalities mainly included surgery alone (*n* = 7), radiotherapy alone (*n* = 33), concurrent chemoradiotherapy (*n* = 17), surgery combined with current chemoradiotherapy (*n* = 32), and surgery plus radiotherapy (*n* = 49).

**Results:**

The median follow-up time for the entire cohort was 61 months (range, 4–231 months). The 5-year overall survival (OS), locoregional failure-free survival (LRFFS), and distant metastasis-free survival (DMFS) rate were 69.6, 78.0 and 73.9%, respectively. Surgery combined with radiotherapy elicited superior survival results, and the combination of surgery and current chemoradiotherapy achieved the best prognoses for all patients, patients with advanced Kadish disease, patients receiving intensity modulated radiation therapy and those with positive surgical margin. Univariate analysis identified orbital invasion and treatment modalities were predictors for OS, LRFFS and DMFS. Lymph node metastasis was associated with OS and DMFS, but not LRFFS. Intracranial invasion, advanced Kadish stage and not receiving concurrent chemotherapy were also predictive of lower OS. Multivariate analyses indicated that lymph node metastasis was an independent prognostic factor affecting DMFS, whereas treatment modalities was independent prognostic factors for OS and LRFFS.

**Conclusion:**

Orbital invasion, intracranial invasion, lymph node metastasis and advanced Kadish disease at initial diagnosis were significantly associated with inferior prognosis. Regarding the treatment modality, the optimal strategy remined surgery with radiotherapy-based multimodality treatment. The concurrent chemoradiotherapy may play a more beneficial role.

## Background

Esthesioneuroblastoma (ENB) is an uncommon malignant neoplasm which accounts for only 3 to 6% of all cancers in the nasal cavity and paranasal sinuses. It has been generally believed that ENB arises from basal cells of the olfactory neuroepithelium within the superior nasal vault [1–3]. ENB was first described by Berger, et al. in 1924 [4]. Despite its longstanding recognition, no consensus regarding its uniform staging system and optimal treatment strategy has been reached. The most widely used classification was proposed by Kadish et al. in 1976 [5]. In this classification, stage C was defined as tumor spread beyond the nasal cavity and paranasal sinuses. As the result, a large spectrum of patients with different prognosis classified as group C disease. Given the rarity of this disease, the optimal treatment strategies were mainly derived from single-institution retrospective studies or population-based analyses. Surgery combined with radiotherapy was frequently considered as the standard of care in the previous studies and the role of chemotherapy remained controversial [6–9].

In this retrospective study, we aimed to evaluate the efficacy of different treatment strategies and the potential prognostic factors of patients with non-metastatic ENB.

## Materials and methods

### Patient data

Between April 1984 and December 2018, 138 patients with non-metastatic ENB were retrospectively analyzed. All diagnoses of ENB were based on microscopic and immunohistochemical findings. Disease in all patients was reclassified according to the Kadish stage on the basis of surgical records, clinical documents and imaging findings, which included X-radiography, computed tomography (CT) or magnetic resonance imaging (MRI) images, and 133 patients underwent CT or MRI.

In term of the 138 patients, 91 patients were male and 47 were female. The median age at diagnosis was 36 years (range,7–82 years). With respect to the Kadish stage, the distribution of patients with stage A, B and C was 1, 25 and 112, respectively. Additionally, 34 patients (24.6%) presented with cervical lymph node metastasis (LNM) at diagnosis by physical and radiographical examination. Orbital invasion and intracranial invasion were present in 71 (51.4%) and 47 (34.1%) patients, respectively.

The study was approved by the Cancer Hospital Chinese Academy of Medical Sciences Reviewing Board and adhered to the tenets of the Declaration of Helsinki. Moreover, a waiver for individual patients’ consent was also obtained from this committee due to the retrospective nature of the study.

### Treatment

The treatment modalities mainly included surgery alone (S alone), which was performed in 7 patients, radiotherapy alone (RT alone), which was performed in 33 patients, concurrent chemoradiotherapy (CCRT), which was performed in 17 patients, surgery combined with radiotherapy, which was performed in 81 patients (preoperative in 12 patients and postoperative in 69 patients). Of the 81 patients receiving combined modality therapy, 32 patients were managed with concurrent chemotherapy (S + CCRT), while 49 were not (S + RT). The clinical characteristics were listed in Table 1.

**Table 1:** The characteristics of patients

	S + CCRT	S + RT	CCRT	RT	S	χ^2^	p value
Age							
≤ 60	30 (94%)	43 (88%)	15 (88%)	31 (94%)	1 (14%)	1.575	0.813
> 60	2 (6%)	6 (12%)	2 (12%)	2 (6%)	6 (86%)		
Gender							
Male	22 (69%)	31 (63%)	10 (59%)	22 (67%)	6 (86%)	1.878	0.758
Female	10 (31%)	18 (37%)	7 (41%)	11 (33%)	1 (14%)		
Kaddish							
A/B	5 (16%)	14 (29%)	2 (12%)	5 (15%)	0 (0%)	5.726	0.221
C	27 (84%)	35 (71%)	15 (88%)	28 (85%)	7 (100%)		
Orbital invasion							
Yes	15 (47%)	17 (35%)	12 (71%)	22 (67%)	5 (71%)	12.446	0.014
No	17 (53%)	32 (65%)	5 (29%)	11 (33%)	2 (29%)		
Intracranial invasion							
Yes	14 (44%)	9 (18%)	7 (41%)	13 (40%)	4 (57%)	9.173	0.057
No	18 (56%)	40 (82%)	10 (59%)	20 (60%)	3 (43%)		
LN							
Positive	8 (25%)	5 (10%)	5 (29%)	16 (49%)	0 (0%)	18.104	0.001
Negative	24 (75%)	44 (90%)	12 (71%)	17 (51%)	7 (100%)		
Surgical margin							
Positive	21 (66%)	23 (47%)	–	–	6 (86%)	5.343	0.069
Negative	11 (34%)	26 (53%)	–	–	1 (14%)		

*LN* lymph node, *S* surgery *CCRT* Concurrent chemoradiotherapy, *RT* radiotherapy

Surgical resection was performed in 88 patients. Forty-three patients received open surgery, while 45 patients were treated with endoscopic surgery. Radical surgery with total macroscopic resections was achieved in 38 patients, while positive surgical margin was noted in 50 patients. No cases with N0 disease were managed with elective neck dissection at their surgical treatment.

Of the 131 patients receiving radiotherapy, 56 were treated with two-dimensional radiotherapy (2DRT), 4 patients with three-dimensional conformal radiotherapy (3DCRT) and 71 with intensity modulated radiation therapy (IMRT). The radiation dose depended on the treatment modalities and the combination and sequence of surgery and radiotherapy. For definitive radiotherapy, the median radiation dose was 70 Gy (range: 60–80 Gy). As to pre-operative radiotherapy, the median dose was 50Gy (range: 50–60 Gy). Regarding to post-operative radiotherapy, the median dose was 70 Gy (range: 50–78 Gy) depended on the surgical margin statuses. The dose fractionation of radiotherapy ranged from 2.0 to 2.24Gy (median dose 2.0Gy). Ninety-eight patients treated with neck irradiation of 40–80 Gy include 34 with N-positive disease.

Generally, chemotherapy was administered in selected cases with large volume indicated by imaging examination or with positive margin after surgery. In this study, concurrent chemotherapy was applied in 49 patients. The majority of patients (*n* = 39) were delivered with single-agent cisplatin 25–60 mg/m2 weekly for 4–7 cycles include two cases received concurrent nimotuzumab 200 mg weekly for 5 cycles. The other regimens consisted of cisplatin 80–100 mg/m2 every 3 weeks for 2–3 cycles (*n* = 4), lobaplatin 20 mg weekly for 4–5 cycles (*n* = 4) or paclitaxel 50 mg weekly for 5 or 6 cycles (*n* = 2). Neoadjuvant and adjuvant chemotherapy with the regimen generally consisted of the combination of cisplatin and etoposide were delivered in 44 and 26 patients, respectively.

### Statistical analyses

Overall survival (OS) was calculated as the time from the first day of diagnosis to the date of death or last clinical follow-up. Loco-regional failure-free survival (LRFFS) and distant metastasis-free survival (DMFS) were measured from the date of initial diagnosis to the documented first failures. All statistical analyses were carried out using SPSS software (version 22.0, SPSS, Chicago, IL, USA). The survival rate was calculated by the Kaplan–Meier method, with curve comparisons using the log-rank test. Differences between patient characteristics were analyzed using chi-square test for categorical variables. Univariate analysis was performed using the log-rank test. Multivariate analysis using the Cox proportional hazard model was applied to identify factors with prognostic value from amongst the potential outcome indicators. Differences were considered significant for *p* values < 0.05.

## Result

**Fig. 1:**
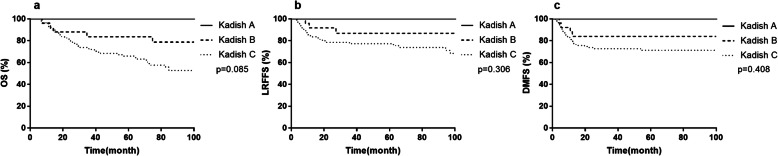
The overall survival rate (**a**), locoregional failure-free survival rate (**b**) and distant metastasis-free survival rate (**c**) among various Kadish stage groups

The median follow-up time for the entire cohort was 61 months (range, 4–231 months). 53 of 138 (38.4%) patients died during follow-up. For all patients, the 5-year OS, LRFFS and DMFS rate were 69.6, 78.0 and 73.9%, respectively. According to Kadish staging system, the 5-year OS, LRFFS and DMFS rates were all 100% for stage A, 83.6, 86.8 and 84.0% for stage B, and 65.9, 75.6 and 71.2% for stage C (*p* > 0.05, Fig. 1).

### Survival by treatment modality

**Fig. 2:**
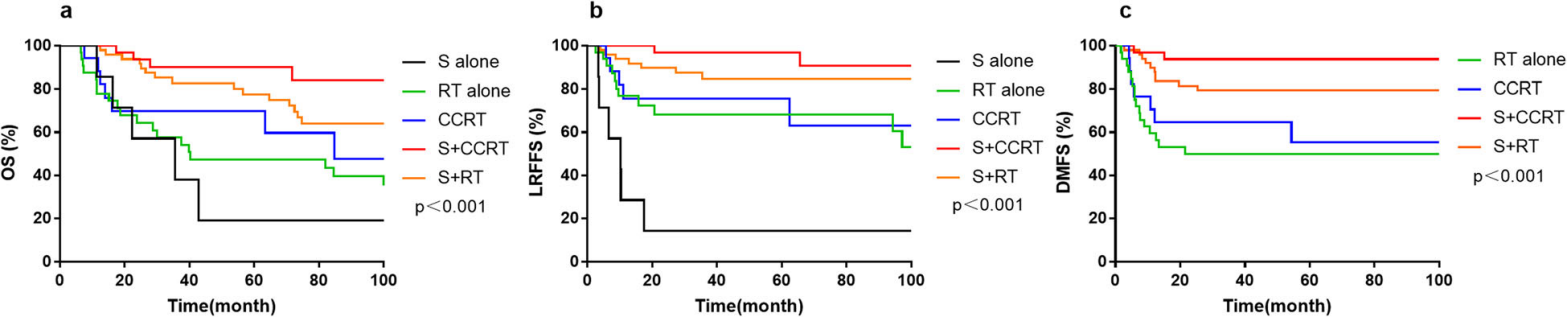
The overall survival rate (**a**), locoregional failure-free survival rate (**b**) and distant metastasis-free survival rate (**c**) among different treatment modalities

Compared with single modality therapy, the combination of modality therapy elicited superior OS (80.2% vs. 43.1%, *p* <  0.001), LRFFS (85.3% vs. 57.7%, p <  0.001) and DMFS (80.0% vs. 49.9%, p <  0.001). In order to evaluate the optional treatment modality for ENB, patients were divided into different groups according to the use of surgery, radiotherapy and concurrent chemotherapy. In the entire cohort, the 5-year OS and LRFFS rates were 90.1 and 96.7% for S + CCRT group, 77.6 and 84.7% for S + RT group, 69.7 and 75.6% for CCRT group, 47.4 and 68.2% for RT alone group, and 19.0 and 14.3% for S alone group. The 5-year DMFS rates for S + CCRT, S + RT, CCRT and RT alone group were 93.8, 79.4, 55.5 and 49.9%, respectively (*p* <  0.001, Fig. 2).

**Fig. 3:**
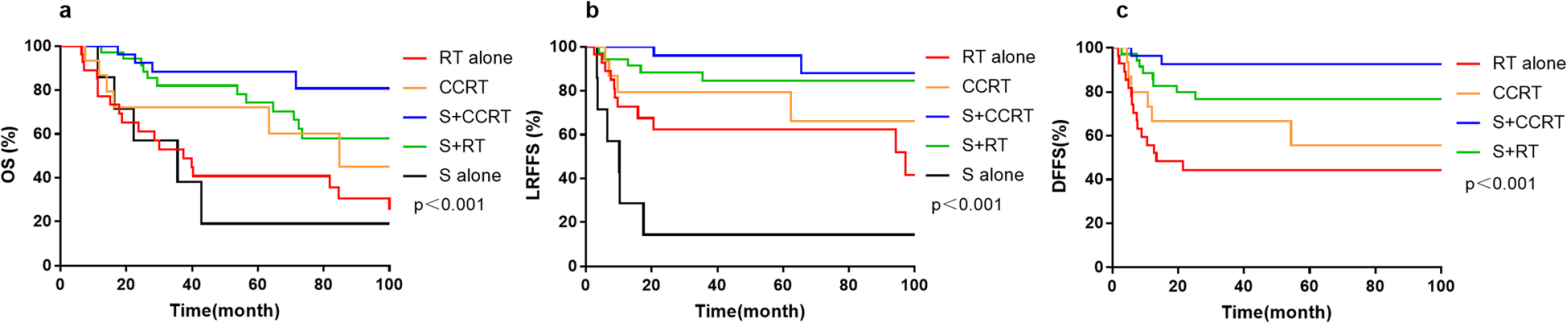
The overall survival rate (**a**), locoregional failure-free survival rate (**b**) and distant metastasis-free survival rate (**c**) among different treatment modalities for patients with Kadish C disease

With respect to the Kadish C stage, the 5-year OS and LRFFS rates were as follows: S + CCRT group, 88.3 and 96.0%; S + RT group, 74.3 and 84.5%; CCRT group, 72.2 and 79.4%; RT alone group, 40.8 and 62.4%; and S alone group, 19.0 and 14.3%. The 5-year DMFS rates for S + CCRT, S + RT, CCRT and RT alone group were 92.6, 76.8, 55.6 and 44.3%, respectively (*p* <  0.001, Fig. 3).

Fifty patients presented with positive margin after surgery, 6 of them refused post-operative RT. These patients who received combined modality therapy demonstrated higher survival rates than patients receiving surgery alone. The 5-year OS and LRFFS rates were 84.9 and 95.0% for S + CCRT group, 67.8 and 81.6% for S + RT group, and 22.2 and 16.7% for S alone group (*p* <  0.05). The DMFS rate was slightly higher in the S + CCRT group compared with S + RT group (90.5% vs 73.4%, *p* = 0.164).

For 71 patients receiving IMRT in recent years, the 5-year OS, LRFFS and DMFS rates were as follows: S + CCRT group (*n* = 28), 88.5, 96.2 and 92.9%; S + RT group (*n* = 17), 74.6, 93.8 and 82.4%; CCRT group (*n* = 17), 69.7, 75.6 and 55.5%; RT alone group (*n* = 9), 44.4, 59.3 and 66.7% (*p* <  0.005).

As to patients with LNM, no one received surgery alone, and no significant differences in 5-year OS, LRFFS and DMFS were noted among the other four treatment modality groups (four groups: S + CCRT, S + RT, CCRT and RT; OS: 58.3, 75.0, 80.0 and 34.4%, *p* = 0.637; LRFFS:85.7, 100, 60.0 and 61.1%, *p* = 0.236; DMFS: 75.0, 60.0, 0 and 42.9%, *p* = 0.276).

### Prognostic analysis

**Fig. 4:**
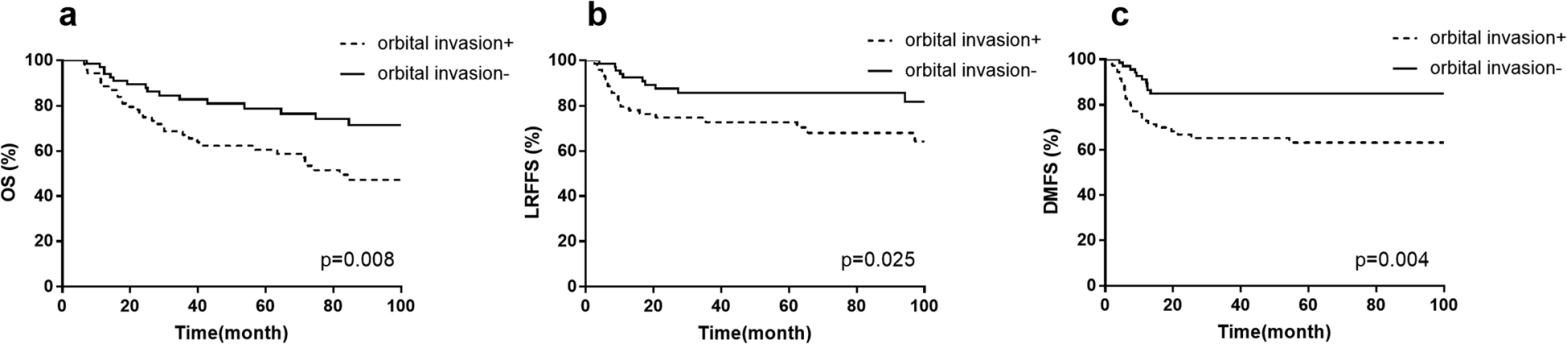
Kaplan-Meier curves of survival according to orbital invasion, (**a**) overall survival, (**b**) locoregional failure-free survival, (**c**) distant metastasis-free survival

**Fig. 5:**
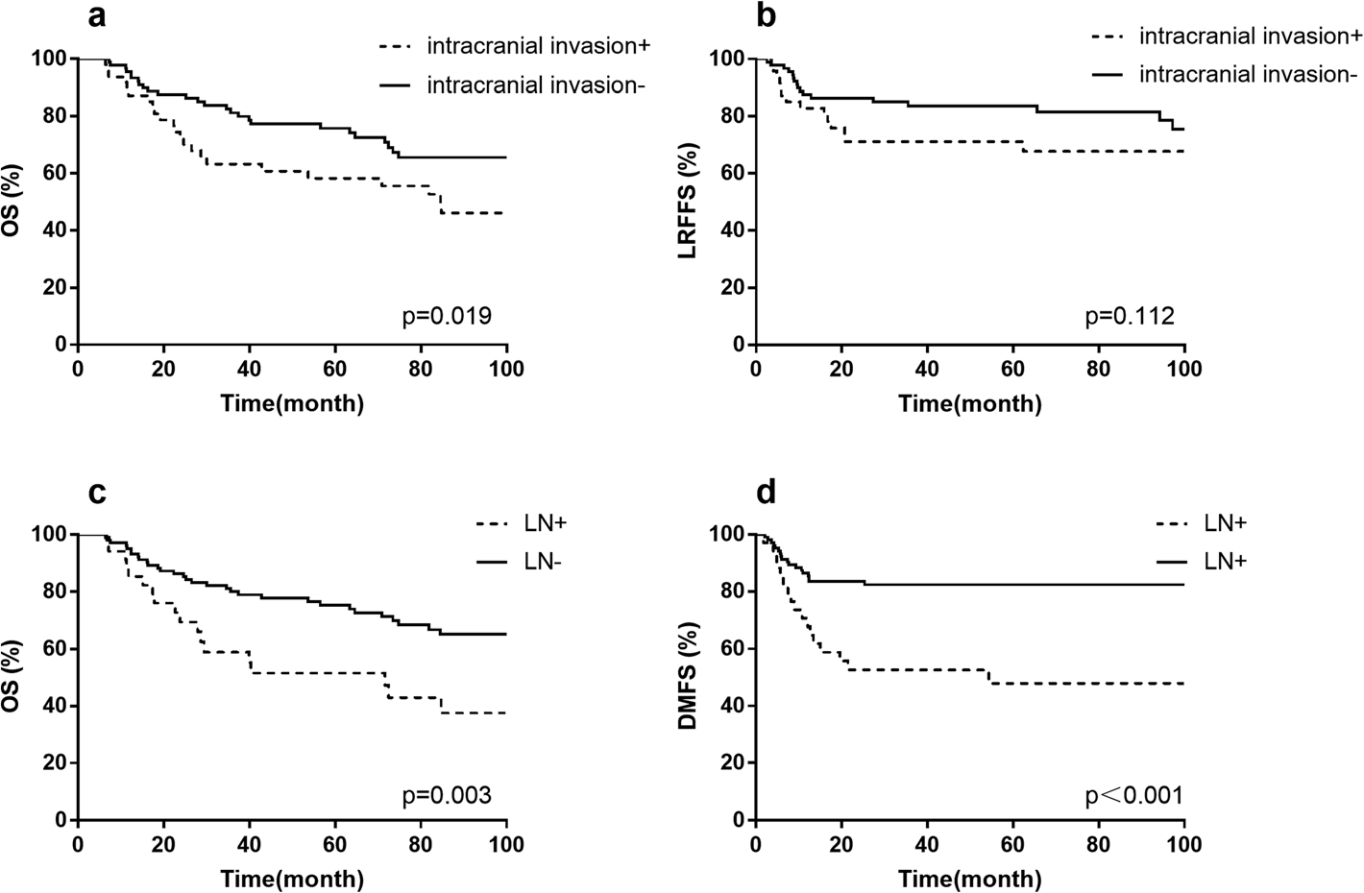
Kaplan-Meier curves of survival according to intracranial invasion, (**a**) overall survival, (**b**) locoregional failure-free survival; according to lymph node status, (**c**) overall survival, (**d**) distant metastasis-free survival

In univariate analysis (Table 2), tumor with orbital extension was noted to be associated with inferior survival results. The 5-year OS, LRFFS and DMFS rates for patients with orbital invasion or not were 60.6, 72.8 and 63.3%, 78.9, 85.8 and 85.1%, respectively (*p* <  0.05, Fig. 4). In addition, patients with orbital invasion were more likely to present with positive surgical margin (χ^2^ = 4.709, *p* = 0.030). Intracranial invasion was predictive of lower OS. Patients with or without intracranial invasion presented a 5-year OS rate of 58.2 and 75.8%, respectively (*p* = 0.019, Fig. 5a). The rate of LRFFS were also slightly lower in patients with intracranial invasion (71.1% vs. 83.6%, *p* = 0.112, Fig. 5b). Positive surgical margin occurred more frequently in patients with intracranial invasion (χ^2^ = 9.657, *p* = 0.002). LNM at diagnosis was significantly associated with OS and DMFS, but not LRFFS. Comparison between patients with and without LNM also exhibited poor OS (51.5% vs 75.2%, *p* = 0.003, Fig. 5c) and DMFS rate (47.8% vs 82.4%, *p* < 0.001, Fig. 5d) at 5-year.

**Table 2:** Univariate analysis results of factors affecting survival

		5-y OS (%)	p value	5-y LRFFS (%)	p value	5-y DMFS (%)	p value
Age	≤60	68.1	.142	79.5	.998	72.8	.387
	> 60	74.0		75.5		84.6	
Orbital invasion	Yes	60.6	.008	72.8	.025	63.3	.004
	No	78.9		85.8		85.1	
Intracranial invasion	Yes	58.2	.019	71.1	.112	75.1	.785
	No	75.8		83.6		73.2	
LN	Positive	51.5	.003	74.1	.116	47.8	< 0.001
	Negative	75.2		80.8		82.4	
Kadish stage	C	65.9	.029	75.6	.134	71.2	.193
	A/B	84.2		87.4		84.6	
Concurrent chemotherapy	Yes	83.2	.027	89.5	.252	80.6	.158
	No	62.4		73.3		70.1	
Radiotherapy	2D	70.1	.748	77.1	.461	69.1	.425
	3D/IMRT	74.0		84.7		75.2	
Surgery margin	Positive	69.7	.179	79.4	.286	83.6	.451
	Negative	84.9		88.8		89.3	
Treatment	S + CCRT	90.1	< 0.001	96.7	< 0.001	93.8	< 0.001
	S + RT	77.6		84.7		79.4	
	CCRT	69.7		75.6		55.5	
	RT alone	47.4		68.2		49.9	
	S alone	19.0		14.3		–	

*LN* lymph node *S* surgery, *CCRT* Concurrent chemoradiotherapy, *RT* radiotherapy

In multivariate analysis, lymph node metastasis was an independent prognostic factor affecting DMFS, whereas treatment modality was a significant independent factor affecting OS and LRFFS. Among the various treatment strategies, surgery and radiotherapy alone was used as a reference, and the S + CCRT group significantly improved the results of OS, LRFFS and DMFS (Table 3).

**Table 3:** Multivariate analysis results of factors affecting survival

	OS		LRFFS		DMFS	
	HR (95%CI)	p	HR (95%CI)	p	HR (95%CI)	p
Kadish stage C vs A/B	1.610 (0.582–4.453)	.358	1.436 (0.400–5.152)	.579	0.663 (0.177–2.483)	.541
LN Y/N	1.581 (0.853–2.931)	.145	1.024 (0.467–2.247)	.952	2.781 (1.320–5.861)	.007
Orbital invasion Y/N	1.505 (0.775–2.926)	.228	1.567 (0.660–3.718)	.309	2.434 (0.978–6.060)	.056
Treatment		.007		.007		.071
CCRTvsRT/S	0.644 (0.277–1.498)	.307	0.595 (0.217–1.632)	.313	0.985 (0.402–2.410)	.973
S + CCRTvsRT/S	0.168 (0.058–0.487)	.001	0.209 (0.069–0.633)	.006	0.145 (0.033–0.633)	.010
S + RTvsRT/S	0.511 (0.264–0.991)	.047	0.268 (0.106–0.681)	.006	0.673 (0.285–1.591)	.367
CCRTvsRT/S	0.644 (0.277–1.498)	.307	0.595 (0.217–1.632)	.313	0.985 (0.402–2.410)	.973

*LN* lymph node, *S* surgery, *CCRT* Concurrent chemoradiotherapy, *RT/S* radiotherapy and surgery alone

## Discussion

ENB is an exceedingly rare malignancy with no randomized controlled clinical trials to evaluate the survival outcomes, and to identify the optimal treatment strategy and prognostic factors. Still, multiple studies based on a single center data have presented their experience and the survival rates of this disease [10–14]. Bell et al. [12] analysed 124 patients’ data from a single institution, the OS and disease-free survival rate were 75 and 60% at 5 years, 55 and 40% at 10 years. Jethanamest et al. [15] reported 311 patients that were identified in the Surveillance, Epidemiology, and End Results (SEER) database, with OS rate of 62.1 and 45.6% at 5 and 10 years, respectively, which was similar to the results of the current study.

To date, there is no evident-based guideline for the treatment of ENB. The most widely accepted treatment approach was multimodality strategy combing surgery with radiotherapy [6–8, 16]. A meta-analysis [16] demonstrated that surgery combined with RT should be the optimum approach for patients with ENB. A large series study [7] consisting of 931 patients from the National Cancer Database (NCDB) indicated that the addition of post-operative radiotherapy to primary surgery significantly improve OS rate, especially for patients with advanced Kadish disease. According to an analysis [8] of 511 patients that were identified in the SEER database, the best OS were obtained for the combination of surgery with radiotherapy. The 5-year OS rates for S + RT, S alone and RT alone group were 73, 68 and 35%, respectively (*p* < 0.01). Compared with single modality therapy, surgery combined with radiotherapy elicited better OS, LRFFS and DMFS in the current study.

Chemotherapy was applied more frequently in advanced stage disease, and in patients with positive surgical margin [7], though it was not considered as the first-line treatment. However, several studies found that it is sensitive to chemotherapy [11, 17, 18], which made it reasonable to suggest combined chemoradiation as an optional approach of ENB. A retrospective analysis reviewed 15 cases from MD Anderson Cancer Center [17], the majority of patients (*n* = 12) received induction chemotherapy with regimen of etoposide and cisplatin, and the response rate achieved 68%.

According to a recent analysis of the SEER database [19], chemotherapy was associated with poor disease-specific survival and OS (*p* < 0.001). The possible reasons were as follows: First, the study consisted of patients who received chemotherapy in the primary treatment, but the information regarding the combination and sequence of chemotherapy with radiotherapy or surgery was unknown. Second, the administration method of chemotherapy included cycles and regimens which may influence the effect of treatment, was also unknown. Furthermore, it was unclear whether there was any selection bias regarding stage between the two groups.

The concurrent chemotherapy was used as a radiosensitizer to augment the therapeutic effect of radiotherapy in many malignant tumors [20]. Xiong et al. [21] analysed the prognosis of patients with different treatment approaches. Surgery with radiotherapy-based multimodality treatment elicited superior OS and disease-free survival rate compared with concurrent chemoradiotherapy and single modality therapy (*p* < 0.001), and surgery combined with radiotherapy and chemotherapy yielded the best survival results. Similarly, univariate analysis demonstrated that not receiving concurrent chemotherapy was significantly associated with poor OS in our results. In multivariate analysis, the S + CCRT group exhibited a significant improvement in OS, LRFFS and DMFS compared with the single modality treatment group. The best 5-year OS, LRFFS and DMFS were also observed in S + CCRT group for patients with Kadish C disease and for those with positive margin. Once tumor extended to invade the pterygopalatine fossa or orbital contents, the achievement of clean margins was a challenge to surgeons. Post-operative concurrent chemoradiotherapy could be considered as a better treatment approach. Moreover, for patients receiving IMRT, S + CCRT achieved the highest survival outcomes, which also means concurrent chemotherapy may play a more beneficial role in era of IMRT. In our series, the addition of concurrent chemotherapy to radiotherapy alone also yielded higher survival results, which suggested that concurrent chemoradiation might be a good alternative method when surgery cannot be performed.

The role of chemotherapy remained uncertain and there was no standard chemotherapy regimen. Based on the survival results from the current and previous studies, as well as the chemo-sensitive of ENB, more relevant researches are worth conducting to reevaluate the role of concurrent chemotherapy. The value of concurrent chemoradiotherapy has been proved in most head and neck cancer [22, 23], it may also have more potential effect on treatment of ENB.

Orbital invasion is common at initial diagnosis in ENB. The incidence was 33% in the single center retrospective study by Song et al. [10], and was 51% in the current study. Previous studies of a large cohort showed that orbital involvement was associated with a poor survival result [10, 24]. Song et al. [10] demonstrated that orbital invasion was significantly associated with poor 5-year survival results (OS 41.9% vs 86.1%, progression free survival 41.9% vs 84.8%, LRFFS 43.2% vs 84.8%, DMFS 41.5% vs 86.1%, *p* < 0.001). Recently, Li et al. [25] found that the orbital invasion in grade II/III (II: Tumor invades the extraconal fat; III: tumor invades any of the following: extraocular muscles, eye globe, orbital apex or optic nerve) elicited inferior outcomes compared with grade I (bone wall erosion). The authors suggested that the prognostic value of the degree of orbital invasion for ENB could be further evaluate in larger cohort studies. According to study by Rimmer et al. [24], multivariate analysis demonstrated that both orbital invasion (*p* = 0.002) and intracranial invasion (*p* = 0.022) were independent prognostic factors for disease-free survival. Similar to these reports, univariate analysis identified orbital invasion was predictors affecting OS, LRFFS and DMFS, while intracranial invasion was predictive for lower OS in our results.

The prognostic value of the orbital and intracranial invasion has been reflected in the American Joint Committee on Cancer (AJCC) classification of nasal cavity and ethmoid sinus tumor, which defined the orbital invasion as T3 disease and intracranial invasion as T4b disease. In the Kadish stage for ENB, tumors extending beyond the nasal cavity and paranasal sinus were designated as group C. These factors also could be taken into consider when modifying the staging system for ENB.

The literature illustrated that the incidence of cervical lymph node metastasis at initial diagnosis ranges from 7% to more than 20% [11, 26, 27]. It has been proven that cervical lymph node metastasis is an important adverse prognosis factor affecting survival. According to a study consisting of 381 patients from the SEER database [27], 33 patients presented with lymph node metastasis. The univariate analysis suggested that the cervical lymph node metastasis was predictors affecting disease-specific survival and OS. Song et al. [10] reported that cervical lymph node metastasis was a significant predictor for poor progression-free survival and OS in both univariate and multivariate analysis, and was predictive of lower DMFS rate in univariate analysis. Similarly, our results also demonstrated that LNM was an independent prognostic predictor of DMFS. In our series, we were unable to evaluate the optimal treatment strategy for N positive disease within limited data. Due to its poor distant metastasis-free survival, chemotherapy may play a more beneficial role.

There are several limitations in the current study. It was a retrospective analysis in design. Due to its rarity, our limited data were collected from a single institution over a 30-year period. Owing to the small number of patients in each group, we were unable to use the propensity score matching to deal with the potential confounders. In addition, the prognosis was complex which may be influenced by treatment modality and patients’ physical status. However, our study evaluated the outcomes among different treatment modalities, and provided some information to make decisions for management with this rare type of cancer. We also evaluated the survival outcomes of 71 patients receiving IMRT, and we thought the results could reflect the treatment effects in recent years of our institution. The combination of surgery and current chemoradiotherapy achieved the best prognoses in our results, and the value of current chemotherapy needed to be verified by additional data sets.

## Conclusion

ENB is an exceedingly rare malignancy. Orbital invasion, intracranial invasion, lymph node metastasis and advanced Kadish disease at initial diagnosis were significantly associated with inferior prognosis. These prognostic factors could be taken into consider when modifying the staging system for ENB. Regarding the treatment modality, the optimal strategy remined Surgery with radiotherapy-based multimodality treatment. The combination of surgery and concurrent chemoradiotherapy could be considered, especially for advanced Kadish disease and for patients with positive surgical margin.

## Data Availability

Unable to upload raw patient data due to patient related private information.

## Abbreviations

ENB: esthesioneuroblastoma
OS: overall survival
LRFFS: locoregional failure-free survival
DMFS: distant metastasis-free survival
CT: computed tomography
MRI: magnetic resonance imaging
LNM: lymph node metastasis
S: surgery
RT: radiotherapy
CCRT: concurrent chemoradiotherapy
2DRT: two-dimensional radiotherapy
3DCRT: three-dimensional conformal radiotherapy
IMRT: intensity modulated radiation therapy
SEER: Surveillance, Epidemiology, and End Results
NCDB: National Cancer Database
AJCC: American Joint Committee on Cancer
